# Tricuspid Annulus Measurements in Severe Tricuspid Regurgitation: Comparative Analysis of Cardiac-Gated Computed Tomography Versus Three-Dimensional Transesophageal Echocardiography

**DOI:** 10.1016/j.shj.2025.100679

**Published:** 2025-06-11

**Authors:** Pranav Chandrashekar, Anas Zaqut, Raluca McCallum, Chara Rydzak, En-Ha Wu, Firas Zahr, Scott M. Chadderdon

**Affiliations:** a Division of Cardiovascular Medicine, Oregon Health and Science University, Portland, Oregon; b Division of Diagnostic Radiology, Oregon Health and Science University, Portland, Oregon

**Keywords:** Cardiac computed tomography, Transesophageal echocardiography, Tricuspid annulus, Tricuspid regurgitation

## Abstract

**Background:**

Treatment options for severe tricuspid regurgitation (TR) require a multimodal analysis of the tricuspid annulus (TA). Cardiac computed tomography (CT) is currently considered the gold standard for annular perimeter measurements, though three-dimensional transesophageal echocardiography (3D TEE) can yield similar results. As such, we sought to determine the accuracy and precision of 3D TEE imaging of the TA perimeter compared to CT imaging in outpatients with severe TR.

**Methods:**

Fifty-five patients were referred for multimodality workup for severe TR that included CT and 3D TEE. The 3D TEE imaging was performed in the mid-esophageal (ME) and transgastric views. A semiautomated software program was used to identify and measure the TA with additional manual optimization by the reader. These 3D TEE measurements were compared to cardiac CT imaging.

**Results:**

Out of 55 patients, 3 were excluded for hiatal hernias and 1 was excluded for severe kidney disease. Fifty-one studied patients had an average age of 76 ± 10 years with 59% female. The 3D TEE analysis of the TA perimeter demonstrated an excellent correlation with CT from the ME view, R = 0.88, and from the TG view, R = 0.86, with an average difference of approximately 8.5% when compared to CT. TEE inter-reader variability was approximately 6%, whereas CT variability was 1.4%

**Conclusions:**

The 3D TEE TA perimeter measurements are accurate when compared to CT with a variability of 8.5%. While CT remains more precise, 3D TEE imaging for TA sizing should be considered a near-equivalent modality to CT.

## Highlights

• Three-dimensional transesophageal echocardiography (3D TEE) semiautomated software allows for accurate and reliable assessment of the tricuspid valve (TV) annulus that is comparable to standard computed tomography (CT) imaging.
• The 3D TEE imaging for tricuspid annulus sizing should be considered a near-equivalent modality to CT tricuspid annulus sizing.
• Prior to transcatheter tricuspid interventions, a 3D TEE assessment of the TV annulus can be one of the tools utilized in understanding valve sizing and right ventricular volume status.

## Introduction

It is well recognized that severe tricuspid regurgitation (TR) has significant consequences for patients, with yearly all-cause mortality estimates ranging from 10% to 40% depending on the associated comorbidities.^1^ Until recently, the only option beyond maximized diuretic therapy and medical therapy for heart failure for patients with either primary or secondary severe TR was high-risk surgical tricuspid valve (TV) repair or replacement, with only a class 2a indication for a surgical TR intervention strategy for the reduction of symptoms and heart failure hospitalizations.^2^ Now, with the Federal Drug Administration’s approval of a novel transcatheter tricuspid valve replacement (TTVR) system (Evoque, Edwards Lifesciences, CA) in February 2024, as well as a specific device for tricuspid transcatheter edge-to-edge repair (T-TEER) (TriClip, Abbott Structural Heart, IL) in April 2024, patients with ≥severe symptomatic TR have new treatment options with data supporting an improvement in symptoms and quality of life that extend beyond medical therapy.^3^^,^^4^

There are many challenging factors that go into the decision-making process in the choice of T-TEER vs. TTVR for each patient, and that can be divided broadly into the following categories: 1) patient-specific risk factors: age, renal function, liver function, diuretic dosing, atrial fibrillation, long-term anticoagulation, left ventricular function, and pulmonary hypertension; 2) right ventricular anatomical features: right ventricular size, depth, and function; papillary muscle location and height; and pacemaker or implantable cardioverter-defribrillator lead; and 3) TV morphological characteristics: degree and location of TR, tricuspid leaflet morphology, scallop size, density of chordal structures, gap width, and imaging quality.^5^ One of the primary determining factors in this complex decision-making process is in the anatomic assessment and sizing of the tricuspid annulus (TA).

Cardiac-gated computed tomography (CT) is regarded as the gold standard for tricuspid annular sizing and can be one of the primary predictors for pursuing a TTVR over T-TEER-based therapies.^6^ It is important, however, to consider that certain patient populations cannot undergo cardiac CT, specifically patients who cannot tolerate the administration of the intravenous contrast material due to significant contrast allergy or severe chronic kidney disease. Additionally, updated echocardiographic software is now available in which a semiautomated approach to quickly analyze the size of the TV annulus can be utilized without the process of a direct three-dimensional (3D) manipulation of echocardiographic data to obtain the point-by-point planimetry of the TV annulus that has been performed in the past.^7^ Thus, the objectives of this study were to evaluate the new GE Vivid E95 platform and the 4D Auto TVQ semiautomated TV software in the assessment of the TV annulus and to determine the accuracy and precision of 3D transesophageal echocardiography (TEE) imaging of the TA perimeter compared to CT-based direct analysis in ambulatory outpatients with severe TR.

## Methods

We performed a single-center prospective analysis of patients with severe TR who were referred to the Oregon Health & Sciences University Heart Valve Clinic Program. Standard of care TEE and cardiac-gated CT angiography of the right ventricle and TV annulus were performed.

The accuracy of the GE Vivid Ultra 4D Auto TV quantification system was assessed by comparing 3D TEE to CT-based TA measurements to assess the overall agreement in annular perimeter. Furthermore, the precision of the GE Vivid Ultra 4D Auto TV quantification system was assessed by comparing annular measurements to assess the inter-reader variability for both 3D TEE and CT when comparing a senior and junior echocardiographer trained in structural heart disease to assess the inter-reader variability for 3D TEE as well as a senior and junior radiologist trained in cardiac CT imaging for tricuspid annular measurements to assess the overall agreement in annular perimeter.

**Figure 1:**
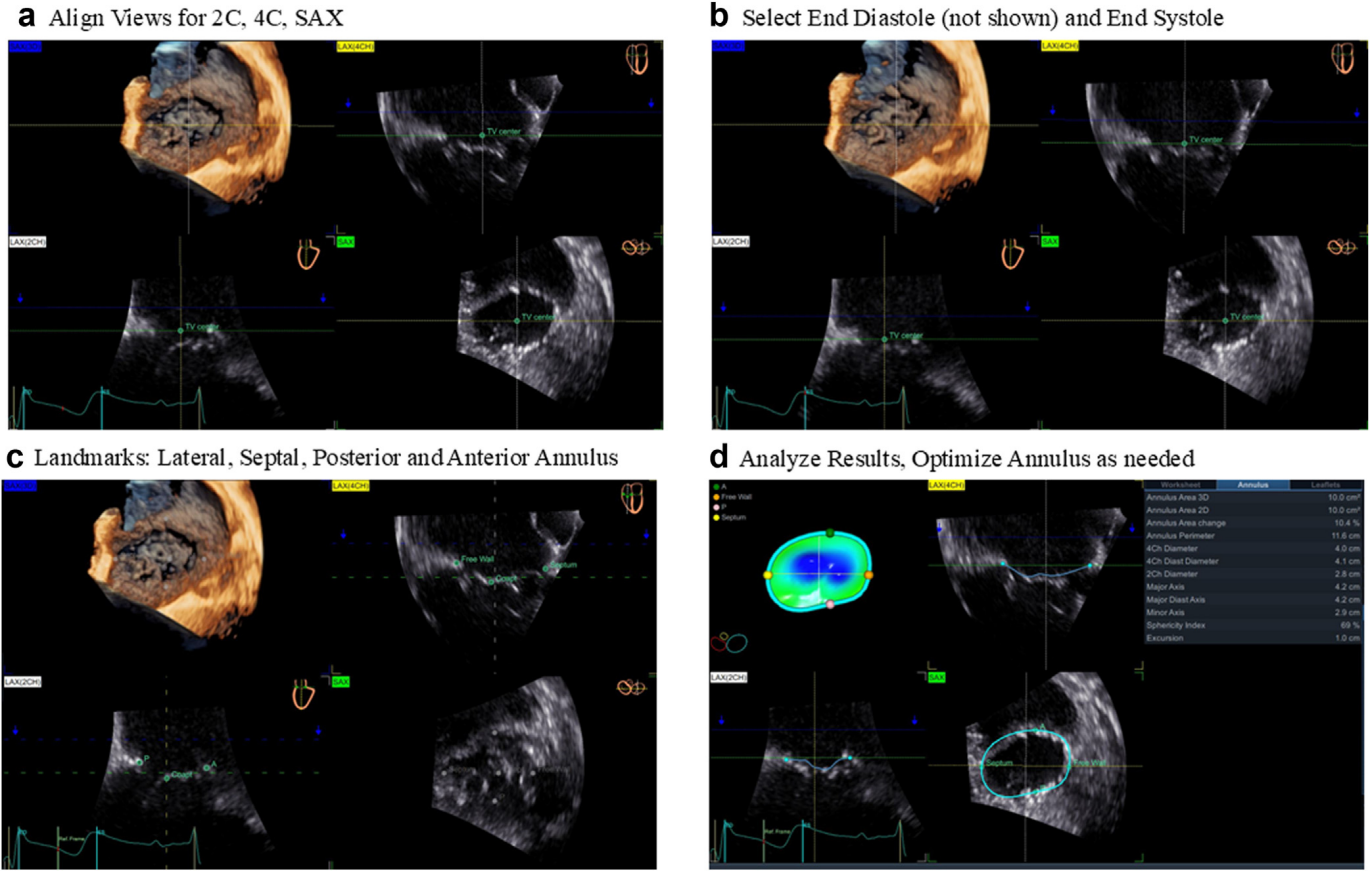
**Tricuspid valve semiautomated quantification steps.** (a) Selection of a 3D volume of the tricuspid valve annulus with a frame rate of ≥20 Hz. The 3D manipulation of the volume to align the 4-chamber (4C) view of the lateral and septal annulus in the upper right quadrant and the 2-chamber (2C) view of the posterior and anterior annulus in the lower left quadrant. (b) Selection of end-diastolic frame (not shown) and end-systolic frame for annulus tracking. (c) Select lateral, septal, posterior, and anterior tricuspid annulus landmarks. (d) Analyze data with annulus analysis displayed on the right side of the right lower quadrant Abbreviation: 3D, three-dimensional; SAX, short axis.

Patients evaluated in the Oregon Health & Sciences University Heart Valve Clinic program for severe TR were referred for a standard of care multimodality workup that included 3D TEE and cardiac CT. The GE Vivid E95 platform and the 4D Auto TVQ semiautomated TV software package were utilized for TEE tricuspid annular measurements. The 3D TEE imaging was performed with a 6VT-D transducer in the mid-esophageal (ME) and transgastric (TG) views with imaging optimized to achieve a frame rate of ≥20 Hz. TA measurements were performed with the 4D Auto TVQ semiautomated TV software package post-TEE procedure either on the GE Vivid E95 system or on a separate GE EchoPACs-enabled computer. After opening the TVQ software package and selecting the appropriate 3D volume image, the echocardiographer identifies end diastole and end systole. The 4D Auto TVQ semiautomated TV software then identifies an early systolic frame in which the operator defines a point on the posterior, anterior, septal, and lateral TA. The software then automatically tracks the TA to generate measurements that can then be manually adjusted as needed in order to optimize the TV annulus tacking and sizing. TA measurements were recorded as perimeter in mm Figure 1.

Gated cardiac CT of the TV was performed on a 320-Multiple Detector Computed Tomography scanner (Aquilion ONE Vision, Canon Medical Systems USA, Inc., Irvine, CA). The scan range covered the entire heart in a cranio-caudal direction. No β-blockers were given. The contrast material (350 mg/mL iodine concentration, iohexol, Omnipaque; GE Healthcare, Chicago, Illinois) was administered at a rate of 4 mL/sec. The total volume of the contrast material ranged from 60 to 80 mL, depending on the patient's body weight. CT images were acquired in a spiral acquisition mode with retrospective electrocardiogram synchronization. The slice thickness of the CT scans was 0.6 mm, and the images were reconstructed with the soft tissue kernel at a slice thickness of 1 mm.

A 3D CT was postprocessed and analyzed on standalone software (Philips Intellispace Portal). Three blinded and independent readers with 1, 3, and 6 years of experience in cardiovascular radiology, respectively, performed the measurement of the TA. Using double-oblique methods, the readers measured the circumference of the TV annulus on the short axis (en face view) using a direct point-to-point fashion in the early systolic phase at 20% of the R-to-R wave interval.

The semiautomated TA perimeter measurements from 3D TEE in both the ME and TG views were compared to early systolic tricuspid annular CT measurements at 20% phase of the cardiac cycle to assess the accuracy of 3D TEE views compared to standard CT views. The precision of TEE and CT was assessed by analyzing the inter-reader variability between senior and junior echocardiographers and radiologists for the respective TEE and CT images.

### Statistical Analysis

All data were analyzed on Prism version 10.1 and are expressed as mean ± SD unless otherwise stated. The D’Agostino-Pearson test was used to assess data normality. Paired CT data were tested for differences with 2-way Student’s *t*-tests, and correlation of parametric data were assessed by Pearson’s product, and nonparametric data were assessed with a Spearman test. Best-fit linear regression analysis was performed when correlations were significant.

## Results

Fifty-five consecutive patients with severe symptomatic TR were consented for the study, and 3 patients were excluded due to hiatal hernias limiting TG imaging, and 1 patient was excluded for severe kidney disease and inability to undergo CT. As such, the final cohort consisted of 51 patients. In general, the average age of the studied cohort was 76 ± 10 years, and 59% were female. Notably, the majority of patients were New York Heart Association Class 3, with 82% having underlying atrial fibrillation or atrial flutter and nearly 25% having a prior surgical mitral valve operation and cirrhotic liver disease noted on CT (Table 1). The average time between CT and TEE was 26 ± 26 days.

**Table 1:** Patient characteristics

Demographics	N = 51
Age (y)	76 ± 10
Sex, female	59
NYHA class	2.8 ± 0.4
LV EF (%)	55 ± 8
Pacemaker	43
Afib/Flutter	82
s/p MVR	24
Hx rheumatic disease	4
Cirrhosis	24
GFR (mL/min/1.73 m^2^)	51 ± 12

Notes. Data presented as % or mean ± standard deviation.

Abbreviations: Afib, atrial fibrillation; GFR, glomerular filtration rate; LV EF, left ventricular ejection fraction; MVR, mitral valve repair/replacement; NYHA, New York Heart Association.

**Figure 2:**
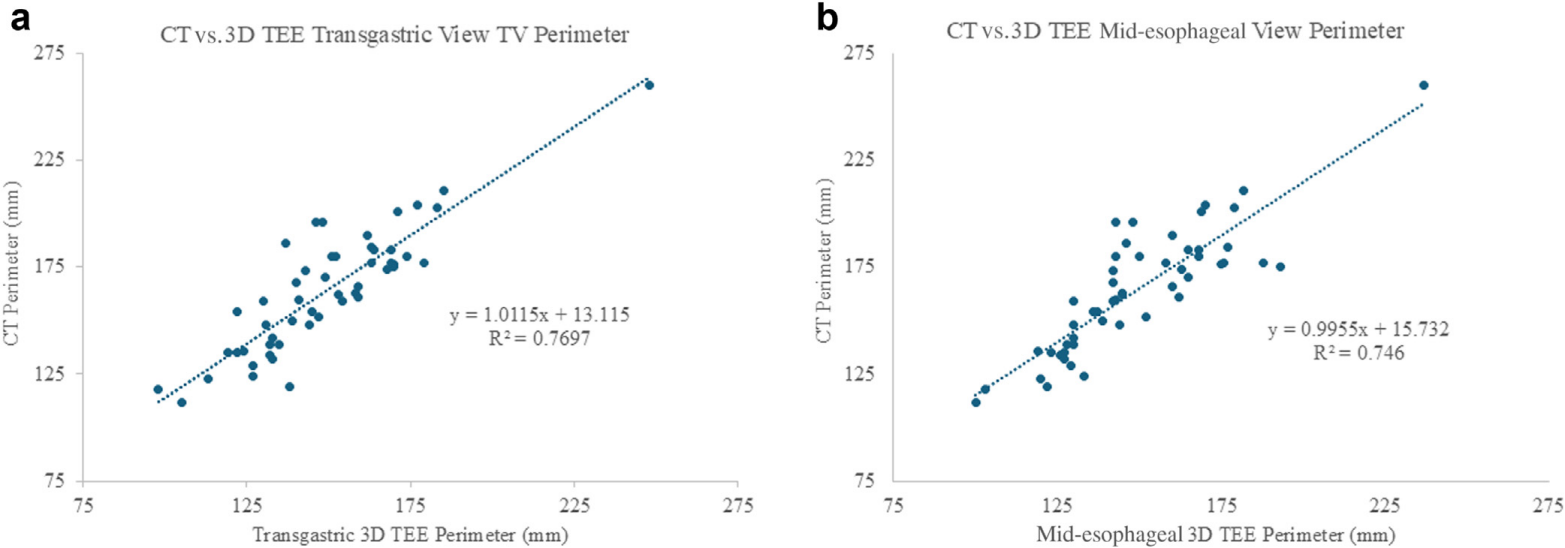
**Accuracy of 3D TEE tricuspid annulus measurements vs. CT.** (a) Correlation of CT vs. 3D TEE from the transgastric view. (b) Correlation of CT vs. 3D TEE from the mid-esophageal view Abbreviations: CT, computed tomography; 3D, three-dimensional; TEE, transesophageal echocardiography; TV, tricuspid valve.

**Figure 3:**
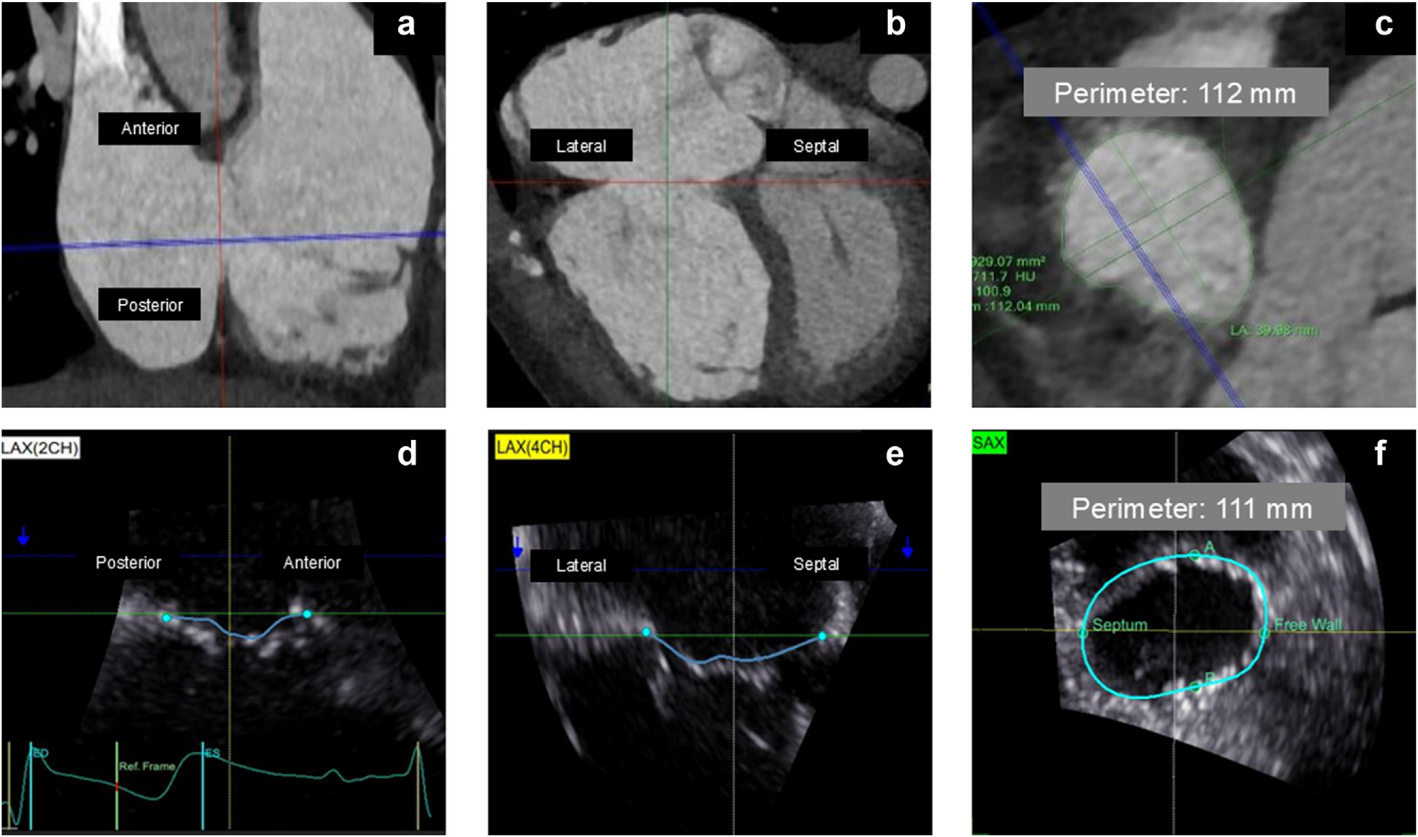
**Representative CT vs. 3D TEE images.** (a) Tricuspid annulus plane in the anterior and posterior view (2-chamber). (b) Tricuspid annulus plane in the lateral and septal view (4-chamber). (c) Tricuspid annulus plane in the en face view with perimeter measurement. (d) The 3D TEE view of the tricuspid annulus plane in the 2-chamber view. (e) The 3D TEE view of the tricuspid annulus plane in the 4-chamber view. (f) The 3D TEE view of the tricuspid annulus plane in the en face view with perimeter measurement Abbreviations: CT, computed tomography; ED, end diastole; ES, end systole; 3D, three-dimensional; LA, long axis; SA, short axis; TEE, transesophageal echocardiography.

The mean TV annulus perimeter of the cohort by 3D TEE from the ME view was 150 ± 26 mm, and the mean TV annulus perimeter by 3D TEE from the TG view was 149 ± 25 mm, with no statistical difference between the 2 imaging views (*p* = 0.96). The mean TV annulus perimeter by CT imaging was 164 ± 28 mm. The average perimeter difference between CT and 3D TEE was 8.5% and was significantly different in a paired analysis compared to each imaging method for 3D TEE (*p* < 0.05). While the measured CT and 3D TEE annulus perimeters were noted to be significantly different, the 3D TEE analysis of the TA perimeter from the TG view demonstrated an excellent correlation with CT with R = 0.88 and 8.5% ± 7.7% difference to CT (Figure 2A). Likewise, 3D TEE analysis from the ME view demonstrated an excellent correlation with CT with R = 0.86 and 8.7% ± 7.7% difference to CT (Figure 2B). For a representative imaging comparison of TV annulus sizing, see Figure 3.

**Figure 4:**
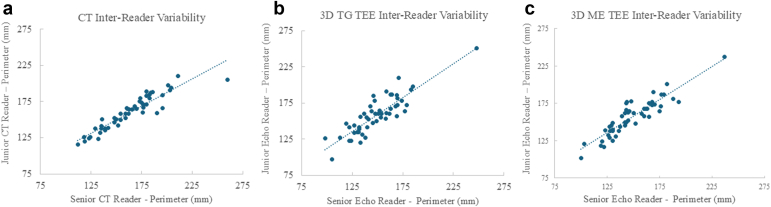
**Precision of 3D TEE tricuspid annulus measurements vs. CT.** (a) Correlation of inter-reader variability of junior vs. senior cardiac imaging radiologists. (b) Correlation of inter-reader variability of junior vs. senior structural imaging cardiologists for 3D TG view. (c) Correlation of inter-reader variability of junior vs. senior structural imaging cardiologists for 3D ME view. Abbreviations: CT, computed tomography; 3D, three-dimensional; ME, mid-esophageal; TEE, transesophageal echocardiography; TG, transgastric.

The precision of CT TV annulus measurements was evaluated by comparing the CT inter-reader variability of a junior radiologist training in cardiovascular imaging compared to a senior cardiac radiologist. The CT inter-reader variability was 1.4% ± 5.7%, R = 0.93 (Figure 4A). Likewise, the precision of 3D TEE TV annulus measurements was evaluated by comparing the TEE inter-reader variability of a junior cardiologist in structural imaging training to a senior structural imaging cardiologist. The 3D TEE inter-reader variability for TG imaging measurements was 6.6% ± 9.3%, R = 0.87 (Figure 4B), and the variability of ME imaging was 5.8% ± 7.4%, R = 0.91 (Figure 4C).

## Discussion

The analysis of the semiautomated, 4D Auto TVQ TV software program on the GE Vivid E95 platform demonstrates excellent correlations of 3D TEE tricuspid annular perimeter measurements obtained from the ME views as well as the TG views when compared to the cardiac CT imaging. Although the correlations between CT and 3D TEE imaging were excellent with strong correlation coefficients, the TA perimeter measured by TEE was statistically different than CT imaging, with an 8.5% smaller annulus perimeter on average. However, there was excellent reliability found between the 3D TEE ME view and the 3D TEE TG view, as there was no significant difference noted in the perimeter measurements, suggesting that either method for obtaining the critical measurement of the TV annular perimeter is valid. Thus, 3D TEE imaging from the ME view or the TG view is an accurate and reliable method of analysis when compared to CT.

The precision of TA measurements clearly favors CT imaging, as the inter-reader variability was on average less than 2%, whereas 3D TEE imaging inter-reader variability was approximately 6%. This can clearly be explained by the inherent differences between the 2 imaging modalities, where CT utilizes venous contrast to help identify the TV annulus and is not limited by respiratory motion or imaging shadows from thoracic and gastric pathology that can limit TEE clarity.

Though the precision of CT is superior to that of 3D TEE, accurate measurements of the TV annulus can still be obtained, as demonstrated here. This is exceedingly important to understand, as cardiologists and cardiac surgeons are now embarking on new preclinical ways and newly released commercial therapies to treat severe TR, where CT annulus sizing is imperative. TTVR is now commercially available therapy for the treatment of severe TR. A primary predictor of success in this procedure is the accurate assessment of the TV annulus perimeter preprocedurally as well as on the day of the procedure. TTVR procedure day 3D TEE imaging is an essential tool to re-evaluate the TV annulus size as one of the ways to ensure appropriate prosthesis sizing compared to the baseline CT obtained during initial TTVR screening. While repeat CT imaging close to a TTVR procedure can be done, this requires additional time, additional radiation, and additional iodinated contrast that can affect renal function. High-quality and reliable 3D TEE imaging and utilization of reliable, semiautomated software programs for a quick analysis of the TV annulus sizing on the day of the procedure are clearly preferable.

A reliable preprocedure assessment of the TV annulus perimeter is a significant factor in the interventional treatment choice when considering T-TEER vs. TTVR for patients with severe TR that remain symptomatic despite diuretic treatment. There is a range of 4 sizes of the Evoque prosthesis, and patients may have an annulus perimeter that is outside the maximum or minimum treatment range that can be reliably identified by 3D TEE, thus potentially limiting the need for an upfront CT strategy until this annulus measurement is obtained. Limiting the use of CT scanning until the 3D TEE tricuspid annulus perimeter has been measured has the potential benefits of reducing iodinated contrast agents and ionizing radiation to patients as well as improving workflows through diagnostic radiology labs by limiting unneeded testing.

There are caveats and limitations of this study to note. First, the GE 4D Auto TVQ semiautomated TV software package tracks the TV annulus in systole and provides an early systolic phase perimeter analysis. Thus, to compare with CT, early systolic phase analysis was performed at 20% of the cardiac cycle. Typically, direct CT and direct TEE analysis of the TA are performed in the diastolic phase when the TV leaflets are at full excursion so the annulus can be identified at leaflet/hinge insertion at its largest diameter. Additionally, while the semiautomated analysis tracks the TV annulus well for both the ME and the TG 3D TEE data, small manual manipulations and editing were performed on each data set to ensure tricuspid annular uniformity.

## Conclusions

The 3D TEE TA perimeter measurements with the GE Vivid system semiautomated TV analysis software are accurate when compared to CT, as demonstrated by the excellent correlations and variability of less than 9%. While CT remains more precise, 3D TEE imaging for TA sizing should be considered a near-equivalent modality to CT and should be favored when CT is contraindicated.

## Review Statement

The review of this manuscript was managed by the guest editor Rahul Sharma, M.D.

## Ethics Statement

The ethics and protocols for this study were approved by the Institutional Review Board of Oregon Health and Science University and conform to the standards of federal regulations, institutional policies, and the Common Rule to ensure protection of rights, welfare, and safety of human subjects.

## Funding

Funding for this study was supported by 10.13039/100006775GE Healthcare, Chicago, Illinois Vivid Ultra E95 Call for Proposals Research.

## Disclosure Statement

S. M. Chadderdon reports financial support was provided by 10.13039/100006775GE Healthcare. The other authors had no conflicts to declare.
